# Acceptance of psychosocial bridging measures in context of dementia

**DOI:** 10.1007/s00702-024-02814-x

**Published:** 2024-08-07

**Authors:** Barbara Leicher, Verena Buschert, Jens Benninghoff, Norbert Scherbaum

**Affiliations:** 1 Clinic for Psychiatry and Psychotherapy, LVR-Klinikum Essen, Clinic and institute of the University, Duisburg-Essen, Germany; 2 Center for Geriatric Medicine and developmental Disorders, kbo-Isar-Amper-Klinikum München-Os, Haar, Germany

**Keywords:** Support measures, Dementia, Cognitive training, Telemedicine, Covid-19

## Abstract

Participants from an outpatient treatment program for cognitive disorders have been offered bridging measures because of limited access to the outpatient clinic during a Covid-19-caused lock-down. The aim of this study was to assess perceived stress, acceptance, and appreciation of the measures among patients and their caregivers compared to the previous bridging measure. Forty participants were offered treatment in person or online depending on their cognitive performance level. To evaluate acceptance, data collected from clinical routine was incorporated into a treatment observation. The evaluation of bridging measures by 25 participants was positive. Perceived stress was moderate to high among participants and has increased significantly compared to previous special treatment. Perceived stress in older patients had increased over the course of the pandemic. Bridging measures represented a treatment alternative and may offer previously untapped potential for location-independent psychosocial treatments in order to ameliorate both the patients’ and their caregivers’ convenience.

**Supplementary Information:**

The online version contains supplementary material available at 10.1007/s00702-024-02814-x.

## Introduction to the topic

From mid-December 2020 to May 2021, Germany was confronted with a second Covid-19-induced lockdown. Rapidly published studies on psychological distress resulting from the extensive restrictions during the first lockdown in Germany (March to June 2020) did not show any impact on individual well-being among the general population (Röhr et al. 2020). With regard to individuals suffering from dementia and their caregivers, experts in the field of gerontology and dementia research urged against subjecting affected individuals to “morbid isolation” and warned of severe consequences (Deutsches Ärzteblatt 2020). As the pandemic progressed, it became evident that the restrictions, although largely supported by older adults (Horn and Schweppe 2020), led to more progressive functional and cognitive decline as well as mental problems particularly in older adults with dementia and among their caregivers (Borges-Machado et al. 2020; Canevelli et al. 2020).

Our clinic offers individuals with acquired cognitive impairment in the context of dementia, as well as their caregivers, a psychosocial outpatient treatment program (further referred to as “Active+++”) consisting of a cognition-focused group intervention, physical activation, and training for caregivers, each lasting 50 min (as suggested by the German guidelines on dementia; DGN e. V. and DGPPN e. V. 2023). During the first lockdown, the treatment program had to be suspended due to the high risk of infection by Covid-19. To reduce stress and continue psychosocial treatment measures, participants were regularly supported by phone and received written exercises on cognition and training by mail. A final evaluation showed that the bridging measure itself, particularly regarding the written exercises, was very well accepted and evaluated, and that the perceived stress was moderate and stable (Buschert et al. 2022). Based on these experiences, the focus of the bridging measures during the second lockdown was on continuing Active+++ with minimal risk of infection, considering group therapy aspects and the different cognitive performance levels of the participants, which also made telemedicine approaches interesting. For individuals with dementia, the feasibility of diagnostics, medical histories, and individual therapies has mainly been assessed in this area (Buschert et al. 2022). The Covid-19 pandemic highlights the need for psychosocial therapy offerings in the context of dementia from distance. Even in the regular, non-pandemic context, these bridging measures may help individuals in rural areas without direct clinic access or who are subject to long-distance car rides to specialized institutions. In this vein, our special bridging measures may be a blueprint to those neglected patients and their caregivers longing for treatment options in the course of dementia.

## Methodology

### Observation design

This was a treatment observation study with two time points (t0-t1) of patients and – if present – their caregivers. In addition, the general concept of the study was linked to bridging measures implemented during the first lockdown as described previously (Buschert et al. 2022). Our outpatient program Active+++ has been suspended again due to the risk of Covid-19 infection on October 16th 2020. Based on feedback from the first bridging measures, a novel program was designed and offered to all participants of our outpatient groups with a two-week pre- and post-preparation phase from January 4th 2021 lasting until the end of the second lockdown on March 27th 2021. Patients received a cognitive intervention four times with 14-day intervals and caregivers got a special training, either face-to-face (individual setting) or online (group setting). Cognitive performance was decisive for the group allocation: Patients with mild to moderate dementia and their caregivers were allocated to individual face-to-face groups, while patients with mild cognitive impairment (Petersen criteria) and their caregivers were assigned to online groups assuming that an online format overloaded the more cognitively impaired people.

Thus, the aim of this treatment observation was verifying the acceptance of the bridging measures, perceived stress of the participants at the beginning and the end and the comparison with the previous treatment observation. For review, standardized self-assessment procedures (Mini Symptom Checklist: Mini-SCL; perceived stress scale: BS) used as part of the usual clinical routine were included and the final evaluation of the measures was recorded using a questionnaire. The study was approved by the responsible ethics commission of the University of Duisburg-Essen (20-9407-BO).

### Observation sample

#### Inclusion criteria and data collection

**Fig. 1:**
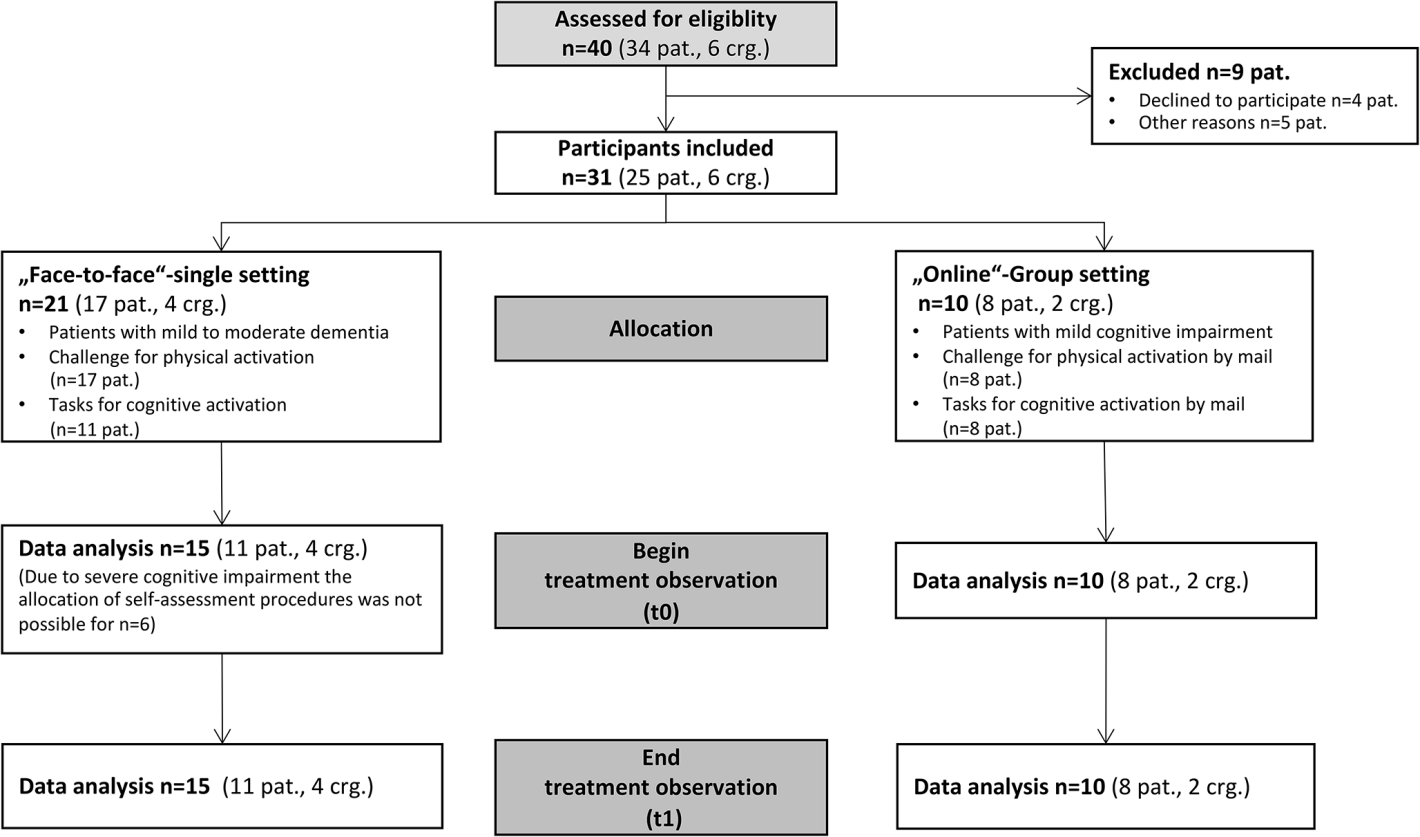
Flow chart of treatment observation

A total of 40 Active+++ participants (34 patients, 6 caregivers), 31 participants (25 patients, 6 caregivers − 6 dyads and 19 patients without caregivers) were included in the bridging program (see Fig. 1). Data was collected at two observation time points in the period from January 4th to March 7th 2021 (t0-t1). Patients in the (mild to) moderate stage of dementia (*n* = 6) participated in the bridging measures. However due to severe cognitive impairment, self-assessment was not possible.

### Face-to-face in presence setting

Patients in the face-to-face setting (*n* = 17) participated in four cognitive stimulation sessions, based on the StaKogS program (Buschert 2017a), each lasting 50 min. Due to pandemic restrictions, both the cognitive intervention and training for caregivers was not performed in groups as usual apart from individual settings. All participants followed the pandemic precautions (FFP2 masks, Covid-19 antigen tests). During each appointment, patients were assigned to “weekly challenges” and they were given instructions for physical activation created by therapists specifically for the home environment.

### Online setting

One week before the first appointment, participants from the online setting group (*n* = 8) were sent written instructions on how the registration and group progress works as well as an invitation code. After participants had received their instructions, the procedure was discussed again by phone and a test-run was carried out. Additionally, patients obtained work sheets for four units of cognitive intervention (StaKogT) by mail (Buschert 2017b). The trainings were conducted online via the Clickdoc software (www.clickdoc.de).

### Training for caregivers

Regardless of the setting of the training for caregivers (face-to-face *n* = 4; online *n* = 2), their program took place in analogue to the regular program and included psychoeducation, strategies for dealing with affected individuals, understanding, and handling changes as well as resource distribution – in the group setting, participants had the chance to exchange their experiences with content adapted specifically to the pandemic.

### Objective parameters

Objectives of the present explorative treatment observation were to capture the perceived stress of the participants during the treatment discontinuation. Further the study aimed to verify the acceptance of the bridging measures and to compare the current and previous treatment observations of the first lockdown.

## Survey tools

When selecting the survey tools, care was taken to ensure that they contain as few items as possible, to allow most of the patients with cognitive impairment to complete them, but as many as necessary to get as much information as possible. To measure perceived stress (BS), a six-stage rating scale was created, which is based on conventional scales (Williamson and Hoggart 2005), in which lower values represent minor levels of stress (1: no stress up to 6: strongest imaginable stress). Additionally, Mini-Symptom-Check-List (Franke 2016) was applied to measure the subjective experiences of discomfort to a total score of 18 items and three subscales (depression, anxiety, somatization) - the higher the value, the stronger the experience of discomfort.

To assess acceptance and evaluation of the bridging measures, a final questionnaire was created. In order not to lose any significance of the questionnaire, in the event of low decisiveness of the participants, an even Likert-Scale was used, so that respondents could either agree with the statements on various thematic areas (“somewhat agree” and “strongly agree”) or reject them (“somewhat disagree” and “strongly disagree”). After the last session the pseudonymized questionnaire was mailed to all participants.

### Data acquisition

As of July 6th 2021, data collected from clinical routine was electronically gathered, evaluated and retrospectively pseudonymized as codes. Personal data could therefore no longer be assigned to a specific person without adding additional information stressing data protection supported by the ethical approval.

### Statistical analyses

Data were processed using the “Statistical Package for the Social Sciences (SPSS)” program, version 25.0 for Windows. Socio-demographic data were compared using non-parametric methods (chi-square; likelihood ratio). For the data analysis of the target variable acceptance of the bridging measures, frequencies of the final questionnaire were calculated using cross tabulations due to small group sizes. For the dependent variable BS, a Mann-Whitney U-test was conducted, and for comparing both bridging measures, the Wilcoxon test was employed. For the Mini-SCL, independent samples t-test was used to discriminate between groups and paired samples t-test for within-group comparison. The independent variables were the group (face-to-face vs. online) and the individual (patients vs. caregivers).

## Results

### Socio-demographic and clinical data

Of a total of 40 participants (34 patients, 6 caregivers) contacted, four patients declined to participate in an online group and five others did not participate due to lack of motivation, resulting in 31 participants included in the final study (25 patients, 6 caregivers). Amnestic data could not be obtained from six patients due to their advanced stage of dementia, therefore data of 25 participants (19 patients and 6 caregivers) were obtained in treatment observation. The average age was 78.35 years (SD = 6.54 years, min = 64, max = 93) and 13 women and 18 men participated. Caregivers were exclusively spouses. Table 1 (electronic supplement) provides an overview of the demographic and clinical data.

**Table 1:** Demographic and clinical data of participants at the beginning of the treatment observation (*n* = 31)

Variable	Group 1 (face-to-face)		Sample group 1 (*n* = 21)	Group 2 (Online)		Sample group 2 (*n* = 10)	Total sample (*n* = 31)	
	Patients (*n* = 17)	Caregivers (*n* = 4)	total	Patients (*n* = 8)	Caregivers (*n* = 2)	total		
**MW (+/-SD)**								
**Age**	79,18 (7,73)	78,25 (5,12)	79 (7,2)	76,88 (5,54)	77,5 (0,71)	77 (4,9)	78,35 (6,54)	
**Perceived stress**	1,9 (1,29)	3,25 (0,96)	2,29 (1,33)	2,71 (1,11)	3 (0,0)	2,78 (0,97)	2,48 (1,2)	
	Patients (*n* = 17)	Caregivers (*n* = 4)	total	Patients (*n* = 8)	Caregivers (*n* = 2)	total	abs. (in %)	
**Gender**	**abs. (in %)**							
Female	7 (41,2)	2 (50)	9 (42,9)	2 (25)	2 (100)	4 (40)	13 (41,9)	
Male	10 (58,8)	2 (50)	12 (57,1)	6 (75)	0 (0,0)	6 (60)	18 (58,1)	
**Marital status**	**abs. (in %)**							
Married	13 (76,5)	4 (100)	17 (81)	7 (87,5)	2 (100)	9 (90,0)	26 (83,9)	
Divorced	0 (0,0)	0 (0,0)	0 (0,0)	1 (12,5)	0 (0,0)	1 (10)	1 (3,2)	
Widowed	4 (23,5)	0 (0,0)	4 (19)	0 (0,0)	0 (0,0)	0 (0,0)	4 (12,9)	
**Level of education**	**abs. (in %)**							
Without a degree	1 (5,9)	0 (0,0)	1 (4,8)	0 (0,0)	0 (0,0)	0 (0,0)	1 (3,2)	
Secondary school leaving certificate	5 (29,4)	1 (25)	6 (28,6)	1 (12,5)	0 (0,0)	1 (10)	7 (22,6)	
Secondary school leaving certificate	5 (29,4)	2 (50)	7 (33,3)	1 (12,5)	2 (100)	3 (30)	10 (32,3)	
Advanced technical college entrance qualification	0 (0,0)	1 (25)	1 (4,8)	0 (0,0)	0 (0,0)	0 (0,0)	1 (3,2)	
General higher education entrance qualification	6 (35,3)	0 (0,0)	6 (28,6)	6 (75)	0 (0,0)	6 (60)	12 (38,7)	
**Status**	**abs. (in %)**							
Participants 1st and 2nd lockdown	11 (64,7)	0 (0,0)	11 (52,4)	5 (62,5)	2 (100)	7 (70)	18 (58,1)	
Participants 2nd lockdown only	6 (35,3)	4 (100)	10 (47,6)	3 (37,5)	0 (0,0)	3 (30)	13 (41,9)	
Severity*	**abs. (in %)**						**Total patients** (*n* = 25)	**Total** **caregivers** (*n* = 6)
Mild cognitive impairment (MCI)				8 (100)	2 (100)	10 (100,0)	8 (32,0)	2 (33,3)
Mild dementia	10 (58,8)	2 (50,0)	12 (57,1)				10 (40,0)	2 (33,3)
Mild to moderate dementia	3 (17,6)	2 (50,0)	5 (23,8)				3 (12,0)	2 (33,3)
Moderate dementia	4 (23,5)		4 (19,0)				4 (16,0)	0 (0,0)

MW = mean value; SD = standard deviation; *for caregivers, refers to the severity level of the patient being cared for by caregivers

### Groups

At the beginning of the treatment observation, there were no significant differences in demographic data (age, gender, marital or educational status) or status (patient vs. relative). As the group allocation of participants was based on the severity level of the patients, there was a significant group difference for both, patients and caregivers (*p* < 0,001) (see Table 2, electronic supplement).

**Table 2:** Differences between groups (face-to-face/online) at the beginning of the treatment observation

	Total sample Group 1 vs. Group 2		
Variable		Patients Group 1 vs. Group 2	Caregivers Group 1 vs. Group 2
Age	t=0.79; *p*=0.44	t=0.75; *p*=0.46	t=0.195; *p*=0.855
Perceived Stress (BS)	t=-0.89; *p*=0.38	t=-1.22; *p*=0.24	t=0.348; *p*=0.745
Gender	χ^2^=0.023; *p*=0.880	χ^2^=0.618; *p*=0.432*	χ^2^=1.50; *p*=0.221*
Marital status	χ^2^=4.071; *p*=0.131*	χ^2^=4.090; *p*=0.129*	all n married
Level of education	χ^2^=3.739; *p*=0.442*	χ^2^=3.554; *p*=0.314*	χ^2^=1.50; *p*=0.472*
Severity**		χ^2^=25.0; *p*<0.001*	χ^2^=6.00;*p*<0.05**

*Likelihood ratio statistic; **for caregivers, refers to severity level of the patient being cared for by caregivers

### Level of perceived stress and discomfort

At the beginning of the treatment observation, there was no significant difference in perceived stress (BS) between the participants or the group, it was moderate to strong for both patients (2.21 ± 1.18) and caregivers (3.17 ± 0.75) and there was no significant change by the end of the measure (*p* > 0.05; see Tables 3 and 4, electronic supplement). With regard to the experience of discomfort (Mini-SCL), there was a significant difference between participants (t(23) = 1.865; *p* = 0.038; d = 5.73 - mean effect size); in patients, discomfort increased significantly by the end of the measures (t(14)=-3.58; *p* < 0.001; d = 6.7 - mean effect size). Concerning the group, there was no significant difference (t(23)=-0.603; *p* = 0.276), both in the face-to-face setting (t(11)=-1.89; *p* = 0.043; d = 7.34 - medium effect size) as well as in the online setting (t(8)=-3.53; *p* = 0.004; d = 4.82 - small effect size) the experience of discomfort increased significantly. The average experience of discomfort by participants at the beginning and end of the bridging measures was in an ordinary range (see Table 4, electronic supplement).

**Table 3:** Overview of the Mann-Whitney test with the dependent (perceived stress) and the independent (group membership and individual) target variable(s)

Timing Variable	t0-t1	Mann-Whitney test
Group	MW=1.32; SD=0.48	U=58.5; Z=-0.95; *p*=0.345
Individual	MW=1.19; SD=0.4	U=30.0; Z=-1.77; *p*=0.076

*mean values ± Standard deviations ( ) are reported

**Table 4:** Overview of perceived stress (BS) and experience of discomfort (Mini-SCL) by individuals (patient/relative) and group affiliation (1=face-to-face, 2=online; *n*=25)

Variable Timing	BS		Mini-SCL	
	Patients Group 1 vs. Group 2	Caregivers Group 1 vs. Group 2	Patients Group 1 vs. Group 2	Caregivers Group 1 vs. Group 2
t0	1.9 (1.29) vs. 2.71 (1.11)	3.25 (0.96) vs. 3.0 (0.0)	2.3 (2.21) vs. 4.86 (6.99)	7.75 (6.4) vs. 6.0 (5.66)
t1	2.09 (0.94) vs. 2.25 (0.71)	2.5 (0.58) vs. 2.5 (0.71)	8.2 (8.84) vs. 11.38 (6.09)	8.5 (3.32) vs. 7.5 (2.12)

*mean values ± Standard deviations ( ) are reported

### Acceptance and evaluation of the bridging measures

Due to the small sample size, only frequencies were reported below; the responses " agree” and “strongly agree” were interpreted as approval and “disagree” and “strongly disagree” as rejection. The final questionnaire was returned by a total of 25 participants. More than 90% of the participants (92.0%) rated the bridging measures as helpful over eight weeks. Over three-quarters of the participants (76.0%) perceived the Covid-19 pandemic as burdensome, with patients expressing more agreement than caregivers (patients: 78.9%, caregivers: 66.7%). The lack of social contacts tended to be greater among caregivers (patients: 73.7%, caregivers: 83.3%). Furthermore patients (68.4%) and caregivers (66.7%) similarly experienced difficulties with restrictions on pursuing hobbies. More than half of the participants denied that existing stress had increased in recent weeks, with a greater emphasis on caregivers (patients: 57.9%, caregivers: 66.7%). The regular visit of Active+++ was missed by almost all patients and half of the caregivers (patients: 94.7%, caregivers: 50.0%). When asked whether the bridging measures were preferred over the regular program almost two thirds (60.0%) of the face-to-face group agreed, while just under a third (30.0%) of the online group disagreed. Regarding the mode of participation, caregivers were more likely to agree (patients: 42.1%, caregivers: 66.7%). Over 80% of the participants (84.0%) stated they would use the program again in a comparable situation, rated work materials (worksheets, “weekly challenges”) as beneficial (84.2%) willing utilize them again (Overview 1, electronic supplement).

### Comparison of the current and previous bridging measure

A total of 18 participants (16 patients, 2 caregivers), with an average age of 78.9 years, of whom 12 were males, participated in both bridging measures. It was not possible to collect self-anamnestic data of four patients due to the advanced stage of dementia. For comparison, perceived stress (BS), experience of discomfort (Mini-SCL) and final assessment of the current (second) and the previous (first) bridging measure were used. In both bridging programs, there were no significant differences in the participants’ perceived stress at the beginning and at the end (all *p* > 0.05; see Table 5, electronic supplement). The experience of discomfort (Mini-SCL), on the other hand, was significantly higher at the end of the second bridging program than before the start and after the end of the first bridging program (see Table 6, electronic supplement). In the final assessment of the bridging measures, there was no significant difference between face-to-face/online training and phone support (t(13) = 1.07; *p* = 0.154).

**Table 5:** Overview of stress experience (BS) and complaint experience (Mini-SCL) by person (patient/caregivers) and bridging measure (*n*=14)

Variable Timing	BS	Wilcoxon test with paired samples	Mini-SCL	t-test for paired samples
t0 bm 1 vs. t0 bm 2	1,79 (0,89)	z=-1.725; *p*=0.084	0,93 (3,87)	t(13)=0.89; *p*=0.193
t0 bm 1 vs. t1 bm 2	1,71 (0,83)	z=-0.905; *p*=0.33	-2,86 (5,59)	t(13)=-1.91; ***p*=0.039**; d=5.59
t1 bm 1 vs. t0 bm 2	2,43 (1,16)	z=-1.724; *p*=0.085	-0,5 (4,13)	t(13)=-0.45; *p*=0.329
t1 bm 1 vs. t1 bm 2	2,07 (0,83)	z=-0.997; *p*=0.319	-4,29 (6,18)	t(13)=-2.59; ***p*=0.011**, d=6.18

*mean values ± standard deviations ( ) are reported; bm= bridging measure

**Table 6:** Overview of Wilcoxon and t-tests for the experience of stress experience (BS) and complaint experience (Mini-SCL) pre/post for participants in both bridging measures (*n*=14)

Variable Timing	BS		Mini-SCL	
	**first bridging measure** patients vs. caregivers	**second bridging measure** patients vs. caregivers	**first bridging measure** patients vs. caregivers	**second bridging measure** patients vs. caregivers
t0	1.67 (0.89) vs. 2.5 (0.71)	2.33 (1.23) vs. 3.0 (0.0)	4.25 (3.5) vs. 4.0 (4.24)	2.83 (2.1) vs. 6.0 (5.66)
t1	1.67 (0.78) vs. 2.0 (1.41)	2.0 (0.85) vs. 2.5 (0.71)	2.5 (3.15) vs. 4.5 (3.54)	7.0 (5.3) vs. 7.5 (2.12)

*mean values ± standard deviations ( ) are reported

## Discussion

The objective of the current bridging measure was to provide the most feasible adaptation of the standard group treatment program, considering hygiene-, distancing-, and contact restrictions during the pandemic, as well as the cognitive performance level of the participants. Once again, it posed a significant challenge to our personal to design and offer bridging measures for individuals affected by dementia within a short timeframe, incorporating insights from the prior treatment observation (Buschert et al. 2022) into the implementation. However, these measures may serve as a blueprint not only for times of pandemic but also for mere regular circumstances of long-distances for patients to reach for stabilizing cognitive training programs.

The survey tools were adapted to the sample by only providing a small number of response options, which may have caused the data collected not to be very differentiated, but it also enabled cognitively impaired patients to self-assess.

We showed significant increase in the subjective experience of discomfort in the present treatment observation is less attributable to a negative intervention effect than to the negative effects of prolonged social isolation and the discontinuation of care and support measures, which other studies also postulated (Cheung and Peri 2020).

The individual face-to-face training was easy to execute and well accepted, from an economic perspective, however, rather unfavorable regarding extensive time and personnel expenditure - similar to the training for caregivers, which made it possible to address personal issues in more detail in an individual setting, which was again also very time-consuming. In conducting online groups, there were various technical difficulties like handling of computers and headphones as well as dialing and logging into the Clickdoc platform. Once all participants were online, the training was easily practicable and according to the participants feedback also an enriching experience. Even some kind of group feeling was created – despite all distance. By considering the feedback from the online group regarding the handling, the targeted group allocation, which based on the assumption that the handling of the online format could lead to excessive demands on more cognitively impaired people, was apparently justified.

A comparison of the bridging measures shows that participants prefer individual or group treatment in person followed by individual treatment by distance (phone/online). The online group set-up performed the worst, as indicated by technical challenges, consistent with findings from other studies (Garcia-Casal et al. 2017). From an economic healthcare perspective, however, online groups would be significantly more economical (in terms of time) compared to individual treatments in person or by phone. By optimizing technical equipment for participants on site, more intensive technical training and a longer adjustment period could increase the acceptance of online treatment.

The results of both treatment observations show that psychosocial bridging measures during the pandemic represented a major benefit for treatment in context of dementia. The Covid-19 pandemic, with its predominantly negative effects, has inevitably prompted considerations within outpatient geriatric psychiatric care regarding alternative treatment methods and experimentation with new technological possibilities. The present explorative treatment observation shows – although only to a limited extent – that telemedical approaches should be considered even at the stage of mild cognitive impairment with adapted, user-friendly technologies. Particularly caregivers who are, for example, unable to participate in outpatient services due to work activities could benefit. In addition, “hybrid groups” consisting of face-to-face and online participation should also be conceivable. In accordance with the demands of the German “National Dementia Strategy” (NDS), both bridging programs can provide positive incentives for location-independent routine care for people with dementia and their caregivers (Federal Ministry for Family Affairs, Senior Citizens, Women and Youth 2020).

### Limitations

The present explorative treatment observation was carried out as part of an outpatient geriatric psychiatric care. The group allocation was based on the severity level of the patient and therefore not randomized, resulting in structural group differences, so that confounding may occur and influence the results accordingly. Due to the small, highly selected sample, it is also difficult to generalize the observations. They may only apply to the sample and situation investigated and may not offer any insights beyond other cases and is therefore not representative. In addition, the small sample size limits the statistical power, making it difficult to detect potential significant differences and effects, or increasing the influence of random variations on the results, potentially distorting them. As the exploratory treatment observation was based on a clinic with a care mandate, there was no control group and therefore no validity with regard to the effectiveness of the bridging measures. Moreover, the pre-post design only allows statements to be made about immediate assessments and evaluations, statements on durability are not possible.

### Electronic supplementary material

Below is the link to the electronic supplementary material.

Supplementary Material 1

## Declarations

### Consequences for clinic and practice

Psychosocial bridging measures regarding dementia in form of a cognitive intervention and training for caregivers in online and face-to-face formats during the Covid-19 pandemic were well feasible and accepted by those affected. Online-based psychosocial interventions also offer opportunities for the participation of patients with mild cognitive impairment and their caregivers from rural areas without direct clinic connections, with mobility restrictions or a lack of logistical options.
