# Supplementary material for: Acceptance of psychosocial bridging measures in context of dementia
Source: J Neural Transm (Vienna). 2024 Aug 7;131(9):1135–42. doi: 10.1007/s00702-024-02814-x (PMC11365855; doi:10.1007/s00702-024-02814-x)
Supplement: Supplementary file 1 — Supplementary Material 1 [file 702_2024_2814_MOESM1_ESM.docx]

**Overview 1** Final evaluation of the bridging measures by group (1=face-to-face, 2=online), n=25

| **Topics and questions from the final questionnaire** | **Group** | **strongly disagree**  abs./in% | **disagree** abs./in% | **agree** abs./in% | **strongly agree** abs./in% |
| --- | --- | --- | --- | --- | --- |
| **Assessment of corona-related changes** |  |  |  |  |  |
| I have perceived the coronavirus pandemic as stressful so far. | 1 (n=15) | 1 (6,7) | 2 (13,3) | 7 (46,7) | 5 (33,3) |
|  | 2 (n=10) | 0 (0,0) | 3 (30,0) | 4 (40,0) | 3 (30,0) |
|  | ***total 25*** | ***1 (4,0)*** | ***5 (20,0)*** | ***11 (44,0)*** | ***8 (32,0)*** |
| I have missed socializing during the coronavirus-related restrictions over the past few weeks. | 1 (n=15) | 0 (0,0) | 2 (13,3) | 5 (33,3) | 8 (53,3) |
|  | 2 (n=10) | 0 (0,0) | 4 (40,0) | 1 (10,0) | 5 (50,0) |
|  | ***total 25*** | ***0 (0,0)*** | ***6 (24,0)*** | ***6 (24,0)*** | ***13 (52,0)*** |
| I have missed being able to pursue hobbies during the coronavirus-related restrictions in recent weeks. | 1 (n=15) | 2 (13,3) | 5 (33,3) | 1 (6,7) | 7 (46,7) |
|  | 2 (n=10) | 0 (0,0) | 1 (10,0) | 2 (20,0) | 7 (70,0) |
|  | ***total 25*** | ***2 (8,0)*** | ***6 (24,0)*** | ***3 (12,0)*** | ***14 (56,0)*** |
| Over the past few weeks, my pre-existing stress levels (physical and/or mental) have increased. | 1 (n=15) | 0 (0,0) | 9 (60,0) | 3 (20,0) | 3 (20,0) |
|  | 2 (n=10) | 1 (10,0) | 5 (50,0) | 2 (20,0) | 2 (20,0) |
|  | ***total 25*** | ***1 (4,0)*** | ***14 (56,0)*** | ***5 (20,0)*** | ***5 (20,0)*** |
| I have missed the regular participation of the Active+++ groups during the coronavirus-related restrictions during the past few weeks. | 1 (n=14) | 0 (0,0) | 1 (7,1) | 5 (35,7) | 8 (57,1) |
|  | 2 (n=10) | 0 (0,0) | 1 (10,0) | 3 (30,0) | 6 (60,0) |
|  | ***total 24*** | ***0 (0,0)*** | ***2 (8,3)*** | ***8 (33,3)*** | ***14 (58,3)*** |
| I perceive the current second lockdown (since December 2020) as more burdensome compared to the first lockdown March-May 2020. | 1 (n=15) | 1 (6,7) | 4 (26,7) | 7 (46,7) | 3 (20,0) |
|  | 2 (n=10) | 0 (0,0) | 5 (50,0) | 1 (10,0) | 4 (40,0) |
|  | ***total 25*** | ***1 (4,0)*** | ***9 (36,0)*** | ***8 (32,0)*** | ***7 (28,0)*** |
| **Bridging training (individual face-to-face, online group)** |  |  |  |  |  |
| I have perceived the regular individual/online training as helpful. | 1 (n=15) | 0 (0,0) | 2 (13,3) | 1 (6,7) | 12 (80,0) |
|  | 2 (n=10) | 0 (0,0) | 2 (20,0) | 2 (20,0) | 6 (60,0) |
|  | ***total 25*** | ***0 (0,0)*** | ***4 (16,0)*** | ***3 (12,0)*** | ***18 (72,0)*** |
| I prefer the individual/online training program to a group in presence. | 1 (n=15) | 1 (6,7) | 5 (33,3) | 4 (26,7) | 5 (33,3) |
|  | 2 (n=10) | 4 (40,0) | 3 (30,0) | 1 (10,0) | 2 (20,0) |
|  | ***total 25*** | ***5 (20,0)*** | ***8 (32,0)*** | ***5 (20,0)*** | ***7 (28,0)*** |
| *Only for group 2:* Participation in the online group program was easy to handle with written/phone instructions when registering. | 2 (n=10) | 1 (10,0) | 1 (10,0) | 3 (30,0) | 5 (50,0) |
| I would engage the individual/online training again in a similar situation. | 1 (n=15) | 0 (0,0) | 1 (6,7) | 2 (13,3) | 12 (80,0) |
|  | 2 (n=10) | 0 (0,0) | 3 (30,0) | 3 (30,0) | 4 (40,0) |
|  | ***total 25*** | ***0 (0,0)*** | ***4 (16,0)*** | ***5 (20,0)*** | ***16 (64,0)*** |
| ***Please only fill in if participation in the Active+++ program before June 2020***  I prefer the individual training/online group offer during the current lockdown to the phone support during the first lockdown (March-May 2020). | 1 (n=8) | 0 (0,0) | 1 (12,5) | 2 (25,0) | 5 (62,5) |
|  | 2 (n=7) | 3 (42,9) | 1 (14,3) | 0 (0,0) | 3 (42,9) |
|  | ***total 15*** | ***3 (20,0)*** | ***2 (13,3)*** | ***2 (13,3)*** | ***8 (53,3)*** |
| **Working materials** |  |  |  |  |  |
| I was able to make good use of the accompanying work materials (e.g. worksheets, exercise instructions for physical activation, "weekly challenges" etc.). | 1 (n=11) | 1 (9,1) | 2 (18,2) | 2 (18,2) | 6 (54,5) |
|  | 2 (n=10) | 1 (10,0) | 0 (0,0) | 2 (20,0) | 7 (70,0) |
|  | ***total 21*** | ***2 (9,5)*** | ***2 (9,5)*** | ***4 (19,0)*** | ***13 (61,9)*** |
| I would want to use working materials by post again in a similar situation. | 1 (n=11) | 1 (9,1) | 2 (18,2) | 1 (9,1) | 7 (63,6) |
|  | 2 (n=10) | 1 (10,0) | 0 (0,0) | 1 (10,0) | 8 (80,0) |
|  | ***total 21*** | ***2 (9,5)*** | ***2 (9,5)*** | ***2 (9,5)*** | ***15 (71,4)*** |
| **Final evaluation** |  |  |  |  |  |
| The Memory Centre's services have helped me during the Covid-19-related restrictions over **the past eight weeks.** | 1 (n=15) | 0 (0,0) | 1 (6,7) | 4 (26,7) | 10 (66,7) |
|  | 2 (n=10) | 0 (0,0) | 1 (10,0) | 1 (10,0) | 8 (80,0) |
|  | ***total 25*** | ***0 (0,0)*** | ***2 (8,0)*** | ***5 (20,0)*** | ***18 (72,0)*** |
